# Goodpasture’s Disease: An Uncommon Disease With an Atypical Clinical Course

**DOI:** 10.14740/jocmr2379w

**Published:** 2015-12-03

**Authors:** Bethel Shiferaw, Viktor Miro, Carroll Smith, Jagadish Akella, Walter Chua, Zae Kim

**Affiliations:** a Department of Medicine, Nassau University Medical Center, East Meadow, NY, USA; b Division of Pulmonary and Critical Care, Nassau University Medical Center, East Meadow, NY, USA; c Division of Nephrology, Nassau University Medical Center, East Meadow, NY, USA

**Keywords:** Goodpasture’s disease, Anti-GBM antibody, Renal failure, Pulmonary hemorrahge, Plasmapheresis, Atypical clinical course

## Abstract

Goodpasture’s disease is an uncommon composite of features including renal failure with pulmonary hemorrhage secondary to an autoimmune response that specifically targets these organ systems. We present a case of particular interest in regards to atypical presentation, and the uncommon treatment that the patient underwent. A 65-year-old Afghani female arrived with complaints of nausea, vomiting, loss of appetite, malaise, decreased urine output, exertional dyspnea, and cough. The patient presented initially with renal failure and unexpectedly developed respiratory failure after hemodialysis. Initial CT of thorax revealed diffuse bilateral pulmonary edema. Subsequently, the patient received a bronchoscopy demonstrating alveolar hemorrhage, which highlights a clinician’s need to maintain a differential and reassess patients. Anti-GBM antibody in the serum was detected and the renal biopsy revealed evidence of the antibody on immunofluorescence. In regards to management, the patient could only be treated with plasmapheresis as she had contraindication to initiation of immunosuppression, after which she showed significant clinical improvement. We would like to highlight the benefit of plasmapheresis without concomitant immunosuppression and recommend such an approach to be considered in similar clinical scenarios, where contraindication for immunosuppressant therapy exists.

## Introduction

Goodpasture’s disease (anti-GBM disease) is a rare autoimmune disease in which antibodies attack the basement membrane in the lungs and kidneys. Goodpasture’s disease may quickly result in permanent lung and kidney damage, often leading to death. The disease was first described by the American pathologist Ernest Goodpasture in 1919 and was later named after him [1].

The estimated incidence of anti-GBM disease is fewer than one case per million population [2]. Anti-GBM disease is responsible for 1-5% of all types of glomerulonephritis and is the cause in 10-20% of patients with rapidly progressive glomerulonephritis [3]. Pulmonary involvement, generally consisting of alveolar hemorrhage leading to respiratory failure, may occur. In rare cases, the pulmonary disease actually predominates the clinical picture [4].

The present case report was selected to emphasize some of the features seen with this uncommon disease and also to illustrate some new ideas in the aspect of therapy.

## Case Report

A 65-year-old Afghan female presented with nausea, vomiting, loss of appetite, malaise and fatigue for 1 week. The patient had noticed decreased urine output for last 3 days, but denied any frequency, urgency or urine color change. The patient has been having dry cough associated with exertional dyspnea. The patient had immigrated to the US 12 years ago. The patient had never smoked nor worked in the coal mines. The patient was never married. The medical history was notable for CAD s/p CABG, hypothyroidism and hypertension.

Physical examination revealed a sick looking female in no acute distress. Her BP was 150/71, P was 108, RR was 18, temperature was 97.7 and oxygen saturation was 97% breathing ambient air. The lungs were clear to auscultation. Cardiac examination was unremarkable. Her abdomen was soft and non-tender with no organomegaly. Her extremities showed no evidence of edema. Neurologic examination was normal.

Laboratory investigation showed a WBC of 9,770/mm^3^, Hgb of 8.4 mg/dL and platelets of 406,000/mm^3^. BUN was 149 mg/dL and serum creatinine was 17.1 mg/dL; a few months ago, serum creatinine was only 3 mg/dL. Blood gases obtained while the patient was on 2 L nasal oxygen were pH: 7.2, PaCO_2_: 18 mm Hg, PaO_2_: 130 mm Hg and bicarbonate level of 7 mg/dL. Urinalysis showed protein of 100 mg/dL, large blood, red cell casts and granular casts. A chest radiograph revealed diffuse bilateral interstitial prominence.

**Figure 1:**
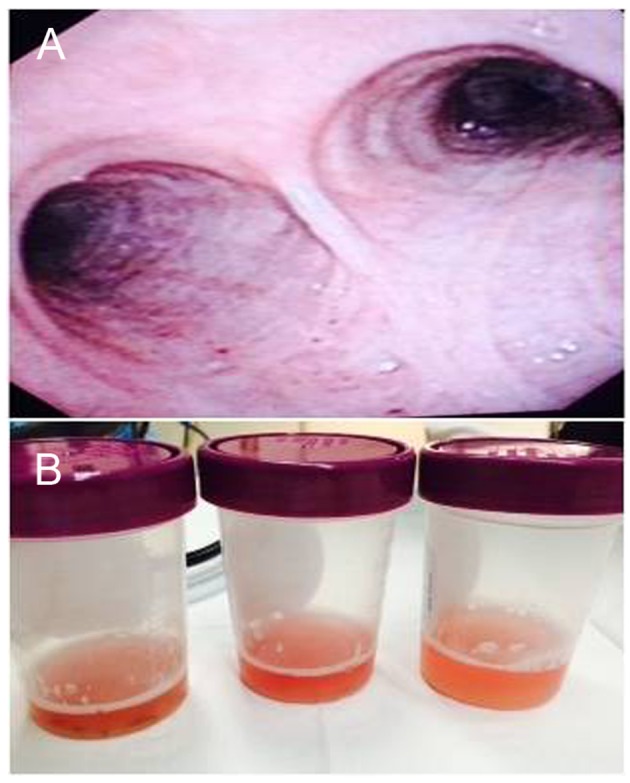
Bronchoscopy with bronchoalveolar lavage of the patient. (A) Bronchoscopy demonstrated intact lung mucosa. (B) Bronchoscopy demonstrated hemorrhagic bronchoalveolar lavage.

The patient was admitted to the hospital for further evaluation. The patient underwent a kidney biopsy and hemodialysis was started urgently for the presenting uremic symptoms. The patient developed progressive worsening of SOB with hypoxemia. HRCT of thorax read as diffuse bilateral peribronchial, interlobular septal, and subpleural thickening most compatible with interstitial pulmonary edema superimposed over the pre-existing fibrosis at both lung bases. Subsequently, the patient received bronchoscopy which highlights a clinician’s need to maintain a broad differential and reassess patient’s clincial course. Bronchoalveolar lavage showed alveolar hemorrhage with clean lung mucosa (Fig. 1).

**Figure 2:**
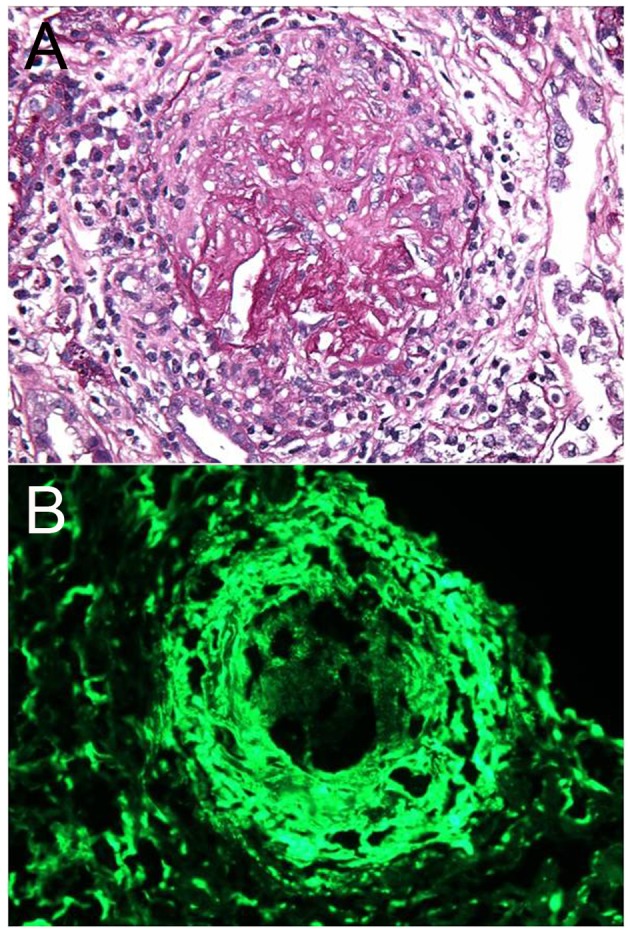
Kidney biopsy of the patient. (A) The light microscopy demonstrated diffuse necrotizing and crescentic glomerulonephritis. (B) The immunofluorescence showed linear staining due to IgG deposition.

Further tests including complement fraction, lupus markers and hepatitis serology were negative. Serology test for proteinase-3 Ab was negative but positive for myeloperoxidase (MPO) Ab. Glomerular basement membrane AB was detected at a level of 1.6 (elevated). Kidney biopsy revealed diffuse necrotizing, crescentic and sclerosing glomerulonephritis. The punctate granular electron densities permeating some compressed glomerular basement membranes and the matrix of the crescent correlate with the immunofluorescence positivity for IgG, kappa and lambda (Fig. 2).

The patient had dropped her hemoglobin multiple times without any overt bleeding during her stay in the hospital. This could be presumed to be secondary to an indeterminate amount of alveolar hemorrhage. The patient was transferred to the ICU with worsening of respiratory failure for a trial of plasmapheresis and steroids (no cyclophosphamide as patient was Quantiferon Gold positive). Patient’s SOB has significantly improved with plasmapheresis and low dose prednisone alone (no cyclophosphamide).

**Figure 3:**
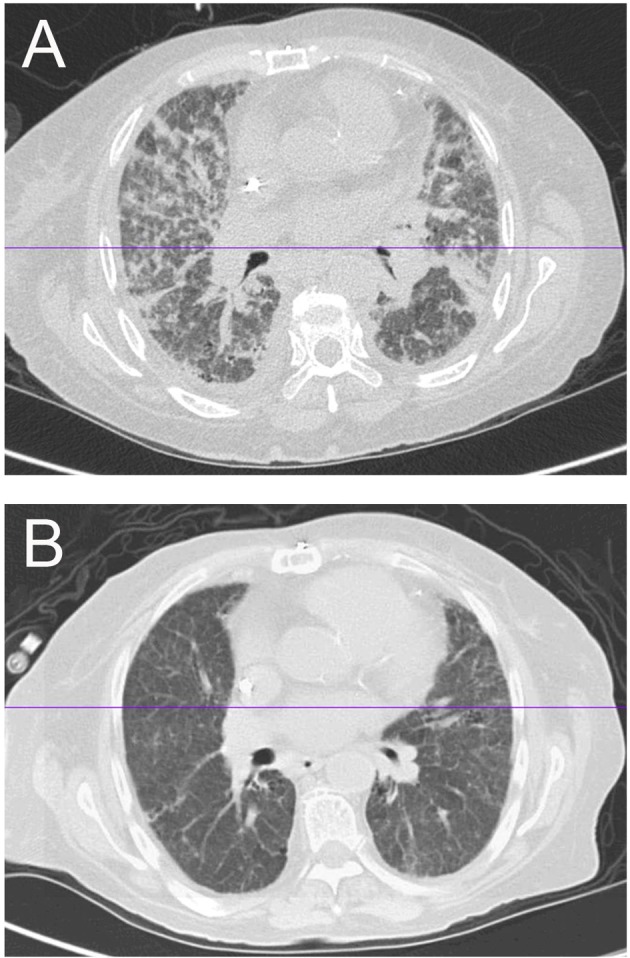
Comparison of imaging before and after treatment (plasmaphoresis). (A) HRCT showing alveolar and interstitial pulmonary edema of bilateral lung fields. (B) CT of thorax showing interval resolution of the alveolar and interstitial pulmonary edema after plasmaphoresis.

Patient finished seven cycles of plasmaphoresis and tapering doses of prednisone. Patient’s symptoms have progressively improved over 4 weeks with repeat anti-GBM antibody negative. Patient’s oxygen requirement has decreased and eventually did not require oxygen for ambulation. Repeat chest X-ray and CT of thorax showed significant improvement after the treatment (Fig. 3).

## Discussion

Goodpasture’s syndrome and Goodpasture’s disease are often used synonymously. The term Goodpasture’s disease is often reserved for those patients with glomerulonephritis, pulmonary hemorrhage, and anti-GBM antibodies. Our patient has positive kidney biopsy, anti-GBM antibody and lung involvement; hence she is diagnosed with Goodpasture’s disease. The discovery of anti-GBM antibodies led to the understanding of the pathogenesis of Goodpasture’s syndrome [5]. These autoantibodies bind to their reactive sites in the glomerular/alveolar basement membranes and activate the complement cascade, resulting in tissue injury. Strong evidence exists that genetics play an important role. The specific human leukocyte antigen type (HLA-DRB1*1501 allele) is considered as a genetic marker for susceptibility to anti-GBM disease [6].

Goodpasture’s disease is extremely rare, with an estimated incidence of 0.5 - 1.8 cases per million per year in European white and Asian populations. The disease predominantly affects the white population, with bimodal age distribution in 20 - 30 years and 60 - 70 years old. The prevalence of the disease is higher in men in the younger age group and women in the older age subgroup. Our patient was an older age Asian female, who belongs to a higher risk category. It is essential to take into consideration the racial, sexual and age variables in patients when entertaining the potential diagnosis of Goodpasture’s. Anti-GBM disease is responsible for up to 20% of acute renal failure due to crescentric glomerulonephritis [7].

Predisposing factors like exposure to hydrocarbons, smoking and infection have been associated with anti-GBM disease. We do not believe that our patient has been exposed to these environmental agents that might have initiated the autoimmune response.

The typical presentation consists of the combination of renal and pulmonary insufficiency. Sixty percent to 80% of patients have clinically apparent manifestations of pulmonary and renal disease, 20-40% have renal disease alone, and less than 10% have disease that is limited to the lungs. Renal manifestations include hematuria, edema, high blood pressure and eventually uremia. Our patient presented with decreased urine output as well as prodromal symptoms, which all are attributed to uremia. The symptoms of pulmonary involvement include cough, shortness of breath, hemoptysis, chest pain and hypoxia, but patients can also be asymptomatic [4]. Our patient initially presented with renal failure and only began to show signs of pulmonary involvement a month later.

Pulmonary symptoms may start weeks to months before renal disease is diagnosed, but pulmonary symptoms may also commence after patients have begun dialysis treatment as well [4]. This is true to our patient’s case as her pulmonary symptoms became more pronounced after she started hemodialysis. Our patient noted shortness of breath as well as non-specific chest pain, sometimes even only hours after dialysis. The EKG had always remained normal and cardiac markers remained unremarkable during these episodes.

The diagnosis of Goodpasture’s disease is made by the detection of anti-GBM antibodies [3]. The enzyme-linked immunosorbent assays (ELISAs) for anti-GBM antibodies are highly sensitive (> 95%) and specific (> 97%). Anti-GBM antibodies, in one-third of the patients, are detected simultaneously with antineutrophilic cytoplasmic antibodies (ANCAs), most frequently myeloperoxidase (MPO-ANCA). This is reflected in our patient by the fact that the serology result detected both antibodies, which makes this case even very rare. A renal biopsy is essential in diagnosing suspected anti-GBM disease, with renal involvement allowing diagnostic confirmation [3]. Light microscopy demonstrates features of a proliferative or necrotizing glomerulonephritis with cellular crescents. Immunofluorescence stains are confirmatory. These show bright linear deposits of immunoglobulin G (IgG), and complement (C3) along the glomerular basement membranes. These typical findings were noted in our patient indicating that she has both serology and tissue confirmatory tests.

The treatment of choice is plasmapheresis in conjunction with cyclophosphamide and prednisone [8]. Plasmapheresis removes circulating anti-GBM antibodies and other mediators of inflammation (such as complement), while the immunosuppressive agents minimize new antibody formation. Plasmapheresis is generally instituted after the diagnosis of Goodpasture’s disease is established either by renal biopsy or by detection of anti-GBM antibodies. Our patient developed worsening respiratory symptoms despite dialysis, which had urged us to perform plasmapheresis. Hence, our patient was started on plasmapheresis and prednisone (1 mg/kg daily) to which the patient responded well. Concern for latent TB prevented us from initiating cyclophosphamide. Plasmapheresis seems to have offered an advantage in the treatment of Goodpasture’s disease via removal of anti-GBM antibodies compared to immunosuppression therapy alone [8]. The extent and duration of plasmapheresis and immunosuppressive therapy is generally not known due to limited clinical trials. Our patient was treated with plasmaphoresis every other day for seven cycles and was able to clear the anti-GBM antibodies.

Untreated anti-GBM disease has an almost universally poor outcome, with death from renal failure or lung hemorrhage [9]. Treatment with plasmapheresis, corticosteroids, and immunosuppressive agents has dramatically improved prognosis, and the 5-year survival rate exceeds 80% [10]. Patients presenting with serum creatinine levels greater than 4 mg/dL, oliguria, and more than 50% crescents on renal biopsy rarely recover. The median survival rate for patients with anti-GBM disease who started renal replacement therapy for end-stage renal disease (ESRD) was estimated to be 5.93 years [11]; unfortunately these data apply to our patient as she had these poor prognostic factors at presentation.

### Conclusion

Goodpasture’s disease is an uncommon cause of renal failure and lung hemorrhage with poor prognosis if not treated, hence prompt diagnosis and treatment is essential for improving clinical outcome. We encountered a patient who presented initially with renal failure and eventually developed respiratory failure due to anti-GBM disease. Our patient was treated with plasmapheresis only as she had contraindication to initiation of immunosuppression (cyclophosphamide), after which she showed significant clinical improvement. We would like to highlight the benefit of plasmapheresis without concomitant immunosuppression and recommend such approach to be considered in similar clinical scenarios, where contraindication for immunosuppressant therapy exists. Anti-GBM disease has been thoroughly studied for many years, and current clinical practices/experiences such as ours could add valuable insight to the fund of knowledge currently available.
